# Quantitative ^18^F-FDG PET-CT can assess presence and extent of interstitial lung disease in early severe diffuse cutaneous systemic sclerosis

**DOI:** 10.1186/s13075-024-03447-x

**Published:** 2024-12-19

**Authors:** Bo Broens, Esther J. Nossent, Lilian J. Meijboom, Gerben J. C. Zwezerijnen, Julia Spierings, Jeska K. de Vries-Bouwstra, Jacob M. van Laar, Conny J. van der Laken, Alexandre E. Voskuyl

**Affiliations:** 1 Department of Rheumatology and Clinical Immunology, Amsterdam UMC, Meibergdreef 9, Room G7-126, Amsterdam, 1105 AZ the Netherlands; 2 Department of Pulmonary Medicine, Amsterdam UMC, Amsterdam, the Netherlands; 3 Amsterdam Cardiovascular Sciences Research Institute, Amsterdam, the Netherlands; 4 Department of Radiology and Nuclear Medicine, Amsterdam UMC, Amsterdam, the Netherlands; 5 Department of Rheumatology and Clinical Immunology, University Medical Center Utrecht, Utrecht, the Netherlands; 6 Department of Rheumatology, Leiden University Medical Center, Leiden, the Netherlands

**Keywords:** Systemic sclerosis, Interstitial lung disease, Positron Emission Tomography, Imaging

## Abstract

**Background:**

This study aimed to assess the quantitative uptake of ^18^F-FDG PET-CT in the lungs of patients with early severe diffuse cutaneous systemic sclerosis (SSc) with and without interstitial lung disease (ILD), compared to controls. In patients with SSc-ILD, ^18^F-FDG uptake was correlated to high-resolution computed tomography (HRCT) and pulmonary function test (PFT) parameters.

**Methods:**

A prospective, cross-sectional study was conducted, involving 15 patients with SSc-ILD, 5 patients with SSc without ILD, and 7 controls without SSc. ^18^F-FDG PET-CT scans were performed following standardized protocols, and quantitative analysis of tracer uptake was conducted in predefined lung regions. In addition, HRCT scans were evaluated for ILD-related radiologic abnormalities. Between-group differences were compared with non-parametric tests, while correlations with PFT parameters were analyzed using Spearman correlation coefficients.

**Results:**

^18^F-FDG uptake was mainly increased in the dorsobasal lung fields of patients with SSc-ILD compared to SSc without ILD and controls (*p* = 0.03 and *p* < 0.001, respectively). ^18^F-FDG uptake was higher in SSc patients with extensive ILD (≥ 20% vs < 20%, *p* = 0.04) and correlated with lower DLCO% (*R* = -0.59, *p* = 0.02). Ground-glass opacities, with or without reticulation, corresponded to increased ^18^F-FDG uptake.

**Conclusions:**

^18^F-FDG PET-CT can detect metabolic activity in the lungs of patients with early severe diffuse cutaneous SSc and ILD, correlating with higher ILD extent (≥ 20%) and lower DLCO%. These results suggest the potential utility of ^18^F-FDG PET-CT in the early detection of ILD (progression) and aiding in risk stratification.

**Supplementary Information:**

The online version contains supplementary material available at 10.1186/s13075-024-03447-x.

## Background

Systemic sclerosis (SSc) is a debilitating auto-immune connective tissue disease, characterized by vasculopathy, inflammation and fibrosis of the skin and internal organs [1]. SSc has a high disease specific mortality, which is estimated at approximately 55%. Interstitial lung disease (ILD) is the leading cause of death, contributing to 35% of SSc-related deaths [2]. In SSc-ILD, scarring of the interstitial space eventually leads to fibrosis, stiffening of the lungs and reduced ability of gas exchange [3]. Patients can experience shortness of breath and/or coughing, and in severe cases, progressive fibrotic ILD can eventually lead to respiratory failure. Clinically relevant ILD develops in 30–40% of patients with SSc, with the majority of cases occurring within the first five years of disease onset [4].

Current treatments for SSc-ILD include anti-inflammatory and/or anti-fibrotic therapies, while a minority of patients with severe diffuse cutaneous SSc (dcSSc) may be eligible for stem cell transplantation [5]. Despite the recent implementation of new therapies such as tocilizumab and nintedanib, most patients do not show improvement of lung function or reversibility of ILD related damage on high-resolution computed tomography (HRCT) [6–8]. Consequently, there is a clinical need for new drugs, tailored treatment strategies, and diagnostic and prognostic tools to enable early intervention in patients at high risk of ILD progression, while avoiding overtreatment in patients with a low risk of progression. Although several risk factors for ILD progression have been identified at population level—such as diffuse cutaneous disease, male sex, older age and positivity for anti-topoisomerase I—the disease course of an individual patient with SSc-ILD remains challenging to predict [9].

At present, diagnosis and monitoring of SSc-ILD is performed using pulmonary function tests (PFTs) and imaging with HRCT. In most (80%) patients with SSc-ILD, HRCT reveals a radiologic pattern of non-specific interstitial pneumonia (NSIP). However, other patterns, such as usual interstitial pneumonia (UIP), lymphocytic interstitial pneumonia (LIP) or combinations thereof may occur as well [10]. Unfortunately, HRCT has limited sensitivity in distinguishing inflammation from (end-stage) fibrosis. For example, previous biopsy studies have shown that ground-glass opacities (GGO) on HRCT might reflect inflammation, interstitial edema, or fine fibrotic remodeling at tissue level. Thus it is unlikely that GGO is merely a reflection of inflammation [11].

Positron emission tomography (PET) is an innovative non-invasive imaging technique with the potential to reflect disease activity and aid in treatment stratification [12]. The most commonly used tracer, ^18^F-Fluorodeoxyglucose (FDG), has broad clinical applications in cancer and various inflammatory conditions [13, 14]. In five small retrospective studies, authors report higher uptake of ^18^F-FDG in the lungs of SSc patients with ILD compared to SSc patients without ILD and healthy controls, while higher uptake correlated with ILD extent and PFT parameters. Furthermore, SSc patients with progressive ILD at follow-up had higher ^18^F-FDG uptake at baseline, indicating its potential utility in the improvement of risk stratification [15–19]. However, prospective studies specifically focusing on SSc-ILD patients with a short disease duration are lacking.

In this study, we investigated ^18^F-FDG PET-CT in patients with early severe dcSSc. Patients with early severe dcSSc are at high risk to develop ILD (progression) and there is a high clinical need to improve outcomes for this specific patient group. As a first step, in this cross-sectional study, we aimed to compare uptake of ^18^F-FDG in the lungs between patients with early severe dcSSc with and without ILD and controls without SSc or ILD. Furthermore, in the patients with ILD, we aimed to correlate uptake of ^18^F-FDG in the lungs to standard of care HRCT (investigating ILD-related radiologic findings) and PFT parameters.

## Methods

### Study design and patient selection

This prospective, cross-sectional study was performed as a sub-study of the UPSIDE study (UPfront autologous hematopoietic Stem cell transplantation vs Immunosuppressive medication in early DiffusE cutaneous systemic sclerosis) [20]. In short, patients with early severe dcSSc were eligible to participate in the UPSIDE study if they had a maximum disease duration of 2 years (from the first non-Raynaud phenomenon), extensive skin involvement (modified Rodnan Skin Score ≥ 15) and/or significant organ involvement (heart, lungs or kidneys). All patients underwent protocolled extensive clinical evaluation at baseline (including PFTs), met the ACR/EULAR classification criteria for SSc, and pulmonary hypertension was ruled-out via right-heart catheterization. The full list of inclusion and exclusion criteria is provided in Additional Data 1. Patients were recruited from the Amsterdam UMC, UMC Utrecht and LUMC Leiden between March 2021 and March 2024, following oral and written informed consent. The medical ethics committees of all participating centers approved the study. Control ^18^F-FDG PET-CT scans from individuals without SSc and without ILD, obtained during regular clinical care for suspected malignancy or follow-up, however showing no abnormalities, were also included. These controls provided oral and written informed consent for the use of their scans and data.

### PET-CT protocol and data analysis

Patients underwent ^18^F-FDG PET-CT scans at baseline, prior to the initiation of the study medication. All scans were performed at the Imaging Center of the Amsterdam UMC, location VUmc (VEREOS, Philips Healthcare and QUADRA, Siemens). ^18^F-FDG PET-CT scans were performed in line with the European Association of Nuclear Medicine procedure guidelines [21]. Patients fasted for at least 6 h before ^18^F-FDG injection and glucose levels of all patients were below < 11 mmol/L. Scans were performed between 55 and 70 min after intravenous injection of ^18^F-FDG, with doses adjusted according to body weight. Non-contrast low-dose CT was used for attenuation correction of PET images and for morphologic co-registration, performed with 120 kV and 30 mAs before emission scanning.

^18^F-FDG PET-CT analysis was conducted using Accurate, an in house PET analysis program [22]. Tracer uptake was assessed semi-quantitatively in hand-placed 2 cm diameter spherical volumes of interest (VOIs) in 22 predefined lung regions: basal (6 dorsal VOIs), mid-level (6 dorsal VOIs, 2 ventral VOIs), and apical lung fields (6 dorsal VOIs, 2 ventral VOIs). Each VOI drawing was performed twice by a trained researcher (BB). Dorsal sites of the basal, mid-level and apical VOIs were pooled to perform further quantitative analysis. Basal–apical and mid-level-apical ratios were calculated by dividing the tracer uptake in the pooled dorsal VOIs of these regions. We report mean and maximum standardized uptake values (SUVmean and SUVmax, respectively). To correct for interindividual differences in tracer distribution, lung SUVmean and SUVmax were divided by SUVmean of the blood pool [23]. A detailed overview of the methods, based on a previous study from our research group, is provided in Additional Data 2 [2].

### HRCT protocol and data analysis

HRCT scans were conducted using high-end multidetector CT scanners at the referring academic medical centers or at the imaging center of the Amsterdam UMC, location VUmc. All patients underwent CT scanning of the chest in the supine position during end‐inspiration, target thin-section helical CT scans of 1.0 or 1.25 mm collimation were obtained and reconstructed using a high-spatial-frequency algorithm. The average time between HRCT and PET-CT acquisition was 15 days. All HRCT scans were centrally scored by an experienced ILD radiologist (LJM) and pulmonologist (EJN), who were blinded for any of the clinical data. HRCT scans were scored for the presence of ILD (yes/no), predominant radiologic ILD pattern, ILD extent and description of ILD-related radiologic findings, based on consensus. ILD patterns were defined according to the 2022 American Thoracic Society / European Respiratory Society guideline [24]. ILD extent was visually estimated, with < 20% defined as limited ILD and ≥ 20% as extensive ILD [10, 25]. ILD-related radiologic findings were defined as: GGO, GGO with signs of reticulation, reticulation, consolidation or honeycombing, respectively [26] and were described in 10 areas: dorsobasal (left and right), dorsal and ventral at mid-level (left and right) and dorsal and ventral at apical level (left and right), corresponding to the 22 VOIs of PET-CT quantification.

### Statistical analysis

All statistical analyses were performed using GraphPad Prism or SPSS. A heatmap was automatically created using GraphPad. Normality was assessed using the D’Agostino-Pearson normality test. Group comparisons were conducted using Mann–Whitney U tests or Kruskal–Wallis tests as appropriate. Spearman’s rank correlation was used to investigate correlations between ^18^F-FDG uptake and PFT parameters. *P*-values < 0.05 were considered statistically significant.

## Results

### Patient characteristics

We included 15 patients with SSc-ILD, 5 patients with SSc without ILD and 7 control patients without SSc or ILD. Baseline characteristics of these patients are summarized in Table 1. In both SSc groups, more males than females were included (SSc-ILD 53.3% and SSc without ILD 60.0%), with a median disease duration of less than one year (SSc-ILD 9.4 ± 18.7 months and SSc without ILD 7.9 ± 3.6 months). Compared to patients with SSc without ILD, those with SSc-ILD were older (age 54.0 ± 33.0 vs 39.0 ± 25.0 years), more frequently received immunosuppressive treatment with mycophenolate mofetil before inclusion (93.3% vs 60.0%) and showed lower diffusion capacity of the lungs for carbon monoxide (DLCO%) (64.0 ± 50.0 vs 71.0 ± 32.2). Control patients were older than both SSc groups (age 64.0 ± 58.0 years). Most patients with SSc-ILD had an NSIP pattern (93.3%) and the minority (33.3%) had an ILD extent ≥ 20%. For the 10 patients with an estimated ILD extent < 20% on HRCT, the distribution was as follows: 5 patients with 5%, 3 patients with 10%, and 2 patients with 15% estimated ILD extent.

**Table 1:** Baseline characteristics

	**SSc-ILD (*n* = 15)**	**SSc without ILD (*n* = 5)**	**Controls (*n* = 7)**
**Age (years)**	54.0 ± 33.0	39.0 ± 25.0	64.0 ± 58.0
**Sex, male**	8 (53.3%)	3 (60.0%)	3 (42.9%)
**Smoking (current, previous)**	8 (53.3%)	3 (60.0%)	3 (42.9%)
**Disease duration (months)**	9.4 ± 18.7	7.9 ± 3.6	-
**modified Rodnan skin score**	21.0 ± 28.0	25.0 ± 12.0	-
**ANA positive**	15 (100.0%)	5 (100.0%)	
- Anti-Topoisomerase I	10 (66.7%)	3 (60.0%)	-
- Anti-RNA polymerase III	0 (0.0%)	1 (20.0%)	-
- Other^a^	5 (33.3%)	1 (20.0%)	-
**Previous therapy**	14 (93.3%)	5 (100.0%)	
- MMF	14 (93.3%)	3 (60.0%)	-
- MTX	0 (0.0%)	1 (20.0%)	-
- CS	2 (13.3%)	4 (80.0%)	-
**Pulmonary function test**			
- FVC%	78.0 ± 36.0	81.0 ± 37.0	
- DLCO%	64.0 ± 50.0	71.0 ± 32.2	
**HRCT pattern**			
- NSIP	14 (93.3%)	-	-
- LIP	1 (6.7%)	-	-
**ILD extent on HRCT (≥ 20%)**	5 (33.3%)	-	-

Data are presented as number (%) and median ± range

*CS* Corticosteroids, *DLCO* Diffusing capacity of the Lungs for Carbon Monoxide, *FVC* Forced Vital Capacity, *HRCT* High Resolution Computed Tomography, *ILD* Interstitial Lung Disease, *LIP* Lymphocytic Interstitial Pneumonia, *MMF* Mycophenolate Mofetil, *MTX* Methotrexate, *NSIP* Non-Specific Interstitial Pneumonia,

^a^Other: Anti-PM/Scl, Anti-RNP, Anti-SSA/Ro

### Quantitative analysis of ^18^F-FDG

**Fig. 1:**
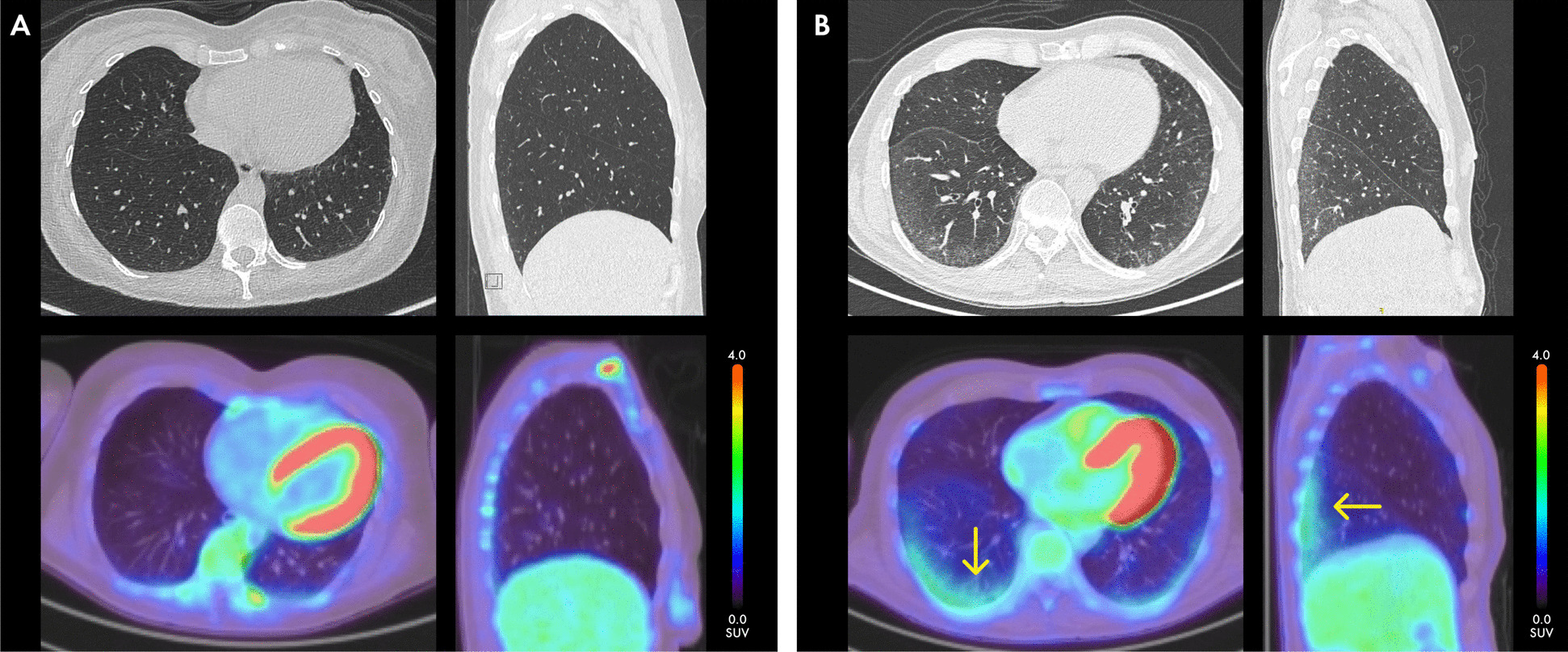
Visual examination of ^18^F-FDG PET-CT and HRCT in SSc-ILD and SSc without ILD. Transverse and sagittal slides of ^18^F-FDG PET-CT and HRCT scans of a SSc patient with (**B**) and without ILD (**A**), ^18^F-FDG uptake is indicated by the orange arrows. ^18^F-FDG = ^18^F-Fluorodeoxyglucose; (HR)CT = (High-Resolution) Computed Tomography; ILD = Interstitial Lung Disease; PET = Positron Emission Tomography; SSc = Systemic Sclerosis; SUV = Standardized Uptake Value

**Fig. 2:**
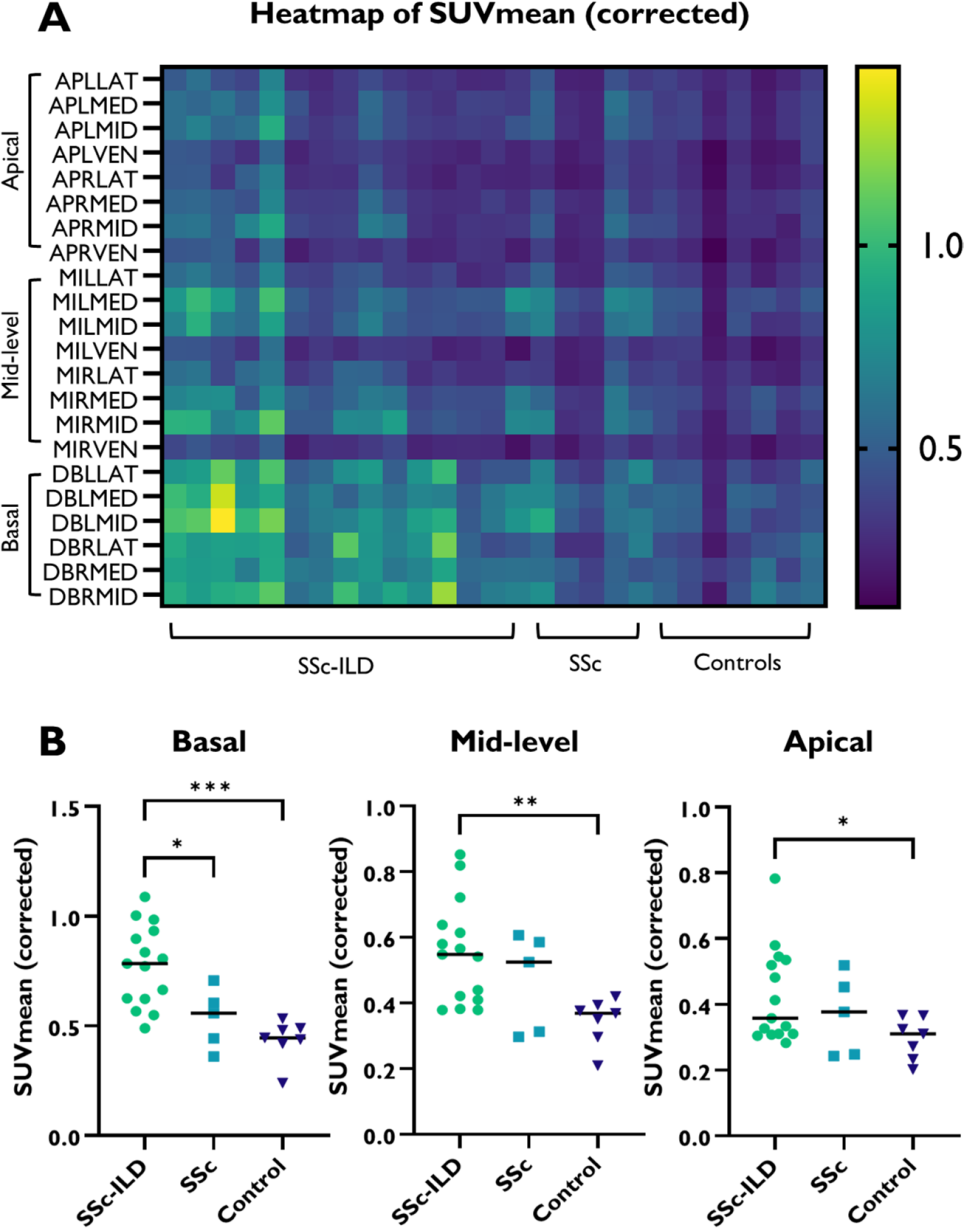
Quantitative pulmonary uptake of ^18^F-FDG in patients with SSc-ILD, SSc without ILD and controls. **A** Heatmap of SUVmean (corrected) in individual VOIs across SSc-ILD, SSc without ILD and controls. **B** Pooled SUVmean (corrected) in the 6 dorsobasal VOIs, 6 mid-level dorsal VOIs and 6 apical dorsal VOIs. * *p* < 0.05 ** *p* < 0.002 ***p < 0.001. ILD = Interstitial Lung Disease; SSc = Systemic Sclerosis; SUVmean = mean Standardized Uptake Value; VOI = Volume of Interest #AP = Apical; MI = Mid-level; DB = Dorsobasal; L = Left; R = Right; LAT = Lateral; MED = Medial; MID = Middle; VEN = Ventral

^18^F-FDG uptake was visually higher in the lungs of patients with SSc-ILD compared to patients with SSc without ILD, which is illustrated in Fig. 1. A heat map, visualizing ^18^F-FDG uptake in individual VOIs per lung level (basal, mid-level and apical) and pooled by group (SSc-ILD, SSc without ILD and controls), clearly shows that the increased uptake was mainly seen in the dorsobasal lung fields (Fig. 2A). Likewise, quantitative analysis showed that uptake of ^18^F-FDG PET-CT in the lungs, as determined by SUVmean, was significantly higher in the dorsobasal lung fields of patients with SSc-ILD compared to patients with SSc without ILD and controls without SSc or ILD (median 0.78 vs 0.56 vs 0.45; *p* = 0.03 and *p* < 0.001, respectively) (Fig. 2B). Interestingly, we observed a trend of higher uptake in patients with SSc without ILD compared to controls. Although differences in ^18^F-FDG uptake were most prominent in the dorsobasal lung fields, significant differences between patients with SSc-ILD and controls without SSc or ILD were also observed in the mid-level dorsal and apical dorsal lung fields (median 0.55 vs 0.37; *p* = 0.002 and median 0.36 vs 0.31; *p* = 0.03, respectively). Furthermore, basal/apical and mid-level/apical ratios were higher in SSc-ILD, when compared to controls without SSc or ILD (median 1.71 vs 1.41; *p* = 0.01 and median 1.32 vs 1.14; *p* = 0.04, respectively). Notably, similar patterns of increased ^18^F-FDG uptake in the lungs were observed when using SUVmax (Additional Fig. 1).

### ^18^F-FDG uptake in relation to ILD extent and ILD-related radiologic findings on HRCT

**Fig. 3:**
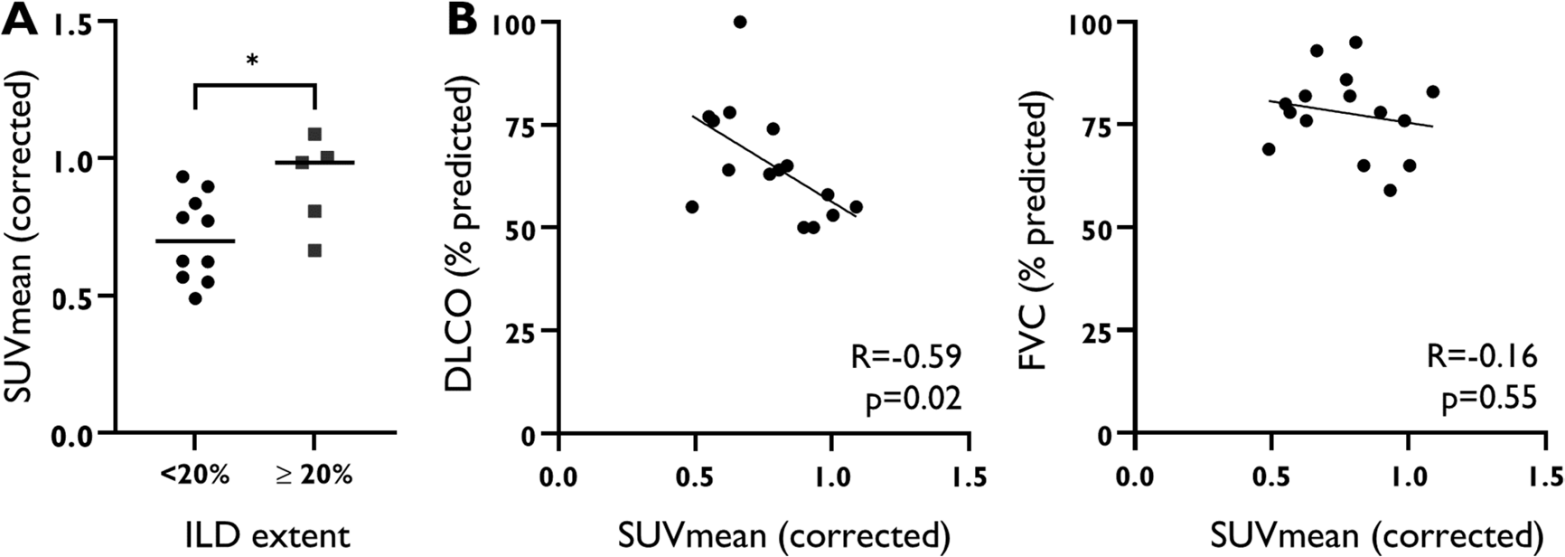
Relation of ^18^F-FDG uptake in the dorsobasal lung fields with ILD extent and pulmonary function test parameters in SSc-ILD. **A** Pulmonary uptake of ^18^F-FDG in SSc-ILD, stratified by ILD extent on HRCT (< 20% and ≥ 20%). **B** Correlation between pulmonary uptake of ^18^F-FDG and pulmonary function test parameters in SSc-ILD. * *p* < 0.05. DLCO = Diffusing capacity of the Lungs for Carbon Monoxide; FVC = Forced Vital Capacity; HRCT = High Resolution Computed Tomography; ILD = Interstitial Lung Disease; SSc = Systemic Sclerosis; SUVmean = mean Standardized Uptake Value

SUVmean in the dorsobasal fields was significantly higher for patients with extensive ILD (≥ 20%; median 0.98) compared to those with limited ILD extent (< 20%; median 0.70 *p* = 0.04)(Fig. 3A). This difference in SUVmean was not observed in the mid-level and apical lung fields. Similar results were noted for SUVmax (Additional Fig. 2).

In patients with SSc-ILD and a NSIP pattern (*n* = 14), we evaluated the presence of ILD-related radiologic findings on HRCT across different lung regions: the left and right site at dorsobasal level, mid-level (dorsal and ventral) and apical level (dorsal and ventral). The dorsobasal lung fields were most affected, with ILD-related abnormalities in all patients and 96.4% of the scored lung fields. GGO was the most common abnormality (71.4%), followed by GGO with reticulation (25%)(Table 2). Medial and apical lung fields, both ventral and dorsal, were affected only in patients with extensive ILD. In these lung fields we mainly observed GGO, whereas reticulation without GGO was noted in just one patient. We did not observe consolidations or honeycombing in any of our patients.

**Table 2:** Radiologic findings and mean SUVmean in the basal, mid-level and apical lung fields in SSc-ILD

	**Basal (dorsal)** **n (%)**	**Mid-level (dorsal)** **n (%)**	**Mid-level (ventral)** **n (%)**	**Apical (dorsal)** **n (%)**	**Apical (ventral)** **n (%)**
Normal	1 (3.6%)	20 (71.4%)	25 (89.3%)	26 (92.9%)	24 (86.7%)
GGO	20 (71.4%)	4 (14.3%)	3 (10.7%)	2 (7.1%)	4 (4.3%)
GGO + Reticulation	7 (25.0%)	3 (10.7%)	0 (0.0%)	0 (0.0%)	0 (0.0%)
Reticulation	0 (0.0%)	1 (3.6%)	0 (0.0%)	0 (0.0%)	0 (0.0%)
	**Basal (dorsal)** **SUVmean**	**Mid-level (dorsal)** **SUVmean**	**Mid-level (ventral)** **SUVmean**	**Apical (dorsal)** **SUVmean**	**Apical (ventral)** **SUVmean**
Normal	0.57 (0.08)	0.51 (0.17)	0.32 (0.10)	0.41 (0.15)	0.32 (0.09)
GGO	0.73 (0.20)	0.70 (0.23)	0.32 (0.07)	0.62 (0.22)	0.38 (0.10)
GGO + Reticulation	0.84 (0.22)	0.58 (0.13)	-	-	-
Reticulation	-	0.61 (0.19)	-	-	-

Data are presented as number (%) and mean (standard deviation)

*GGO* Ground-Glass Opacities, *SUVmean* mean Standardized Uptake Value

In the dorsobasal lung fields, SUVmean in the corresponding VOIs was slightly higher in areas of GGO and reticulation compared to GGO alone, although this difference was not statistically significant (Table 2). However, areas with GGO and reticulation exhibited a significantly higher SUVmean compared to normal lung parenchyma (*p* = 0.03). In the mid-dorsal and apical-dorsal lung fields, SUVmean in areas with GGO was significantly higher than in normal lung parenchyma (*p* = 0.02). These differences were not observed in the ventral lung fields. Results for SUVmax showed a similar pattern, except at the dorsobasal level, where SUVmax was significantly higher in areas with GGO and reticulation compared to areas with GGO alone (*p* = 0.002). Additionally, SUVmax in areas with GGO was significantly higher compared to areas with normal lung parenchyma (*p* = 0.04). Detailed results are provided in Additional Table 1.

### ^18^F-FDG uptake and correlation with PFT parameters

In patients with SSc-ILD, we found a significant negative correlation between DLCO% and SUVmean in the dorsobasal lung fields (*R* = −0.59, *p* = 0.02) (Fig. 3B), indicating that higher ^18^F-FDG uptake is associated with lower DLCO%. A similar correlation was observed for SUVmax (*R* = −0.52, *p* = 0.04; Additional Fig. 2). No significant correlation was found between forced vital capacity (FVC%) and either SUVmean or SUVmax, nor between any of the PFT parameters and SUVmean or SUVmax at mid-level or apical level.

## Discussion

We found that uptake of ^18^F-FDG in the lungs (both SUVmean and SUVmax corrected) was significantly increased in the dorsobasal lung fields of patients with early severe dcSSc and ILD, compared to SSc patients without ILD and controls without SSc or ILD. Additionally, in SSc-ILD, ^18^F-FDG uptake was higher in those with extensive ILD (≥ 20%) and correlated with lower DLCO%. This is the first study to report these findings in a specific group of patients with severe dcSSc, with a short disease duration, and generally limited ILD extent. These findings are of great clinical importance, as they suggest that ^18^F-FDG PET-CT can detect metabolic activity even in early stages, with ILD extent and DLCO% being important markers for ILD progression and mortality [10, 25, 27–29].

Our findings are consistent with previous studies, which have reported higher ^18^F-FDG uptake in SSc-ILD compared to controls, with or without SSc [18, 19]. In these previous studies, the ^18^F-FDG uptake in patients with SSc-ILD was generally even higher, which might be explained by the fact that the included patients had a higher ILD extent. Importantly, we noted a trend for higher uptake in three patients with SSc without ILD compared to controls. Also, we found increased uptake in the mid-level and apical lung fields of patients with SSc-ILD compared to controls, while few ILD-related radiologic abnormalities were found at these levels. These results suggest that even in SSc patients without ILD and in SSc-ILD patients in areas without radiologic abnormalities, there is evidence for increased metabolic activity in the lungs. Follow-up should confirm the meaning of these findings. If these patients develop ILD (progression), these findings might indicate that ^18^F-FDG PET-CT can detect ILD presence/development or progression earlier than is currently possible with HRCT.

When findings of ^18^F-FDG PET-CT were compared and correlated to ILD-related radiologic findings on HRCT, we found that that increased ^18^F-FDG uptake in the dorsobasal lung areas corresponded to the areas most affected on HRCT. Moreover, we observed that ^18^F-FDG uptake coincided with areas exhibiting GGO, with or without reticulation. The increased uptake in GGO, with or without reticulation, together with increased uptake in patients with higher ILD extent aligns with prior studies in SSc-ILD [17, 19] and idiopathic pulmonary fibrosis (IPF) [30–32]. Due to the presence of sole reticulation in only one patient, this data set did not allow conclusions on comparative ^18^F-FDG uptake in these areas as opposed to GGO areas. In particular longitudinal studies will provide additional data to address this investigation.

With regard to PFT parameters, correlations with DLCO% and FVC% have been reported by one retrospective study in SSc-ILD in a cross-sectional setting, while multiple studies in IPF, in which ^18^F-FDG PET-CT has been studied more extensively, have shown correlation with DLCO% and FVC% during follow-up [19, 30, 33–36]. The fact that we did not find any correlation with FVC can be explained by the low prevalence of fibrosis in our patient group, at this stage of the disease.

Despite these promising results, a general concern of ^18^F-FDG is that it has limited specificity for certain disease processes. It is established that uptake of ^18^F-FDG is facilitated by glucose transporters (GLUT). We know from two small histologic studies in IPF and certain pulmonary inflammatory conditions (e.g. chronic pulmonary inflammation, tuberculosis and organizing pneumonia) that GLUT1 is expressed on the surface of erythrocytes and inflammatory cells, while GLUT3 is mainly observed on inflammatory cells. This indicates that ^18^F-FDG uptake may be a reflection of inflammation and neovascularization. However, this has not been formally investigated in SSc-ILD. Furthermore, a possible role for increased aerobic glycolysis in myofibroblasts has been suggested, but has not been confirmed in histologic studies [19, 34, 37]. Thus, new histologic studies, that look into the expression of GLUT in the lungs of patients with SSc-ILD could improve interpretation of ^18^F-FDG PET-CT.

In clinical studies, ^18^F-FDG uptake is increased in different ILD-related radiologic abnormalities (e.g. from GGO to honeycombing), in patients with short and long disease duration and in patients limited and extensive ILD on HRCT [15, 17–19]. Exploration of novel, more specific tracers targeting fibroblast activity, integrins, B-cells and macrophages in clinical studies of SSc-ILD have shown encouraging results [38–42]. In particular, imaging with ^68^Ga-FAPI-04 should be further investigated, as uptake of ^68^Ga-FAPI-04 was higher in SSc patients with ILD progression and changes after follow-up were in line with response to nintedanib [38]. Alongside new tracers, innovative imaging techniques, such as respiratory gating and radiomics, hold potential to improve image resolution, motion correction and PET-CT quantification [43–45].

Strengths of this study include its prospective design and the specific group of patients with early severe dcSSc at high risk of ILD progression or development. Furthermore, results were consistent across different SUV metrics (SUVmean and SUVmax). Limitations of this study include the relatively small patient numbers, which limit regression analysis, and the lack of follow-up. Ongoing follow-up studies, including repeated ^18^F-FDG PET-CT scans approximately one year after baseline and extended clinical follow-up through the UPSIDE study, will be crucial to validate these findings and assess the utility of ^18^F-FDG PET-CT in early detection of ILD (progression), risk stratification, monitoring disease progression and tailored guided treatment strategies.

## Conclusions

In conclusion, ^18^F-FDG uptake, quantified by both SUVmean and SUVmax, is increased in the lungs of patients with early severe dcSSc and ILD, and is related to extensive ILD (≥ 20%) and lower DLCO%. These findings support the potential role of ^18^F-FDG PET-CT in detecting metabolic activity at early stages. Future longitudinal studies are necessary to establish the value of ^18^F-FDG PET-CT over time and its comparative advantage over HRCT and PFTs in this patient population.

## Supplementary Information

Supplementary Material 1.

## Data Availability

The datasets used and/or analyzed during the current study are available from the corresponding author on reasonable request.

## Abbreviations

CT: Computed Tomography
dcSSc: Diffuse Cutaneous Systemic Sclerosis
DLCO: Diffusing capacity of the Lungs for Carbon Monoxide
FDG: Fluorodeoxyglucose
FVC: Forced Vital Capacity
GGO: Ground-Glass Opacities
GLUT: Glucose Transporter
HRCT: High Resolution Computed Tomography
ILD: Interstitial Lung Disease
IPF: Idiopathic Pulmonary Fibrosis
NSIP: Non-Specific Interstitial Pneumonia
PET: Positron Emission Tomography
PFT: Pulmonary Function Test
SSc: Systemic Sclerosis
SUV: Standardized Uptake Value
VOI: Volume Of Interest

## References

[1] Denton CP, Khanna D. Systemic sclerosis. Lancet. 2017;390(10103):1685–99. 28413064 10.1016/S0140-6736(17)30933-9

[2] Tyndall AJ, Bannert B, Vonk M, Airò P, Cozzi F, Carreira PE, et al. Causes and risk factors for death in systemic sclerosis: a study from the EULAR Scleroderma Trials and Research (EUSTAR) database. Ann Rheum Dis. 2010;69(10):1809–15. 20551155 10.1136/ard.2009.114264

[3] Perelas A, Silver RM, Arrossi AV, Highland KB. Systemic sclerosis-associated interstitial lung disease. Lancet Respir Med. 2020;8(3):304–20. 32113575 10.1016/S2213-2600(19)30480-1

[4] Solomon JJ, Olson AL, Fischer A, Bull T, Brown KK, Raghu G. Scleroderma lung disease. Eur Respir Rev. 2013;22(127):6–19. 23457159 10.1183/09059180.00005512 PMC4103193

[5] Vonk MC, Smith V, Sfikakis PP, Cutolo M, del Galdo F, Seibold JR. Pharmacological treatments for SSc-ILD: Systematic review and critical appraisal of the evidence. Autoimmun Rev. 2021;20(12): 102978. 34718159 10.1016/j.autrev.2021.102978

[6] Distler O, Highland KB, Gahlemann M, Azuma A, Fischer A, Mayes MD, et al. Nintedanib for Systemic Sclerosis-Associated Interstitial Lung Disease. NEJM. 2019;380(26):2518–28. 31112379 10.1056/NEJMoa1903076

[7] Highland KB, Distler O, Kuwana M, Allanore Y, Assassi S, Azuma A, et al. Efficacy and safety of nintedanib in patients with systemic sclerosis-associated interstitial lung disease treated with mycophenolate: a subgroup analysis of the SENSCIS trial. Lancet Respir Med. 2021;9(1):96–106. 33412120 10.1016/S2213-2600(20)30330-1

[8] Khanna D, Lin CJF, Furst DE, Goldin J, Kim G, Kuwana M, et al. Tocilizumab in systemic sclerosis: a randomised, double-blind, placebo-controlled, phase 3 trial. Lancet Respir Med. 2020;8(10):963–74. 32866440 10.1016/S2213-2600(20)30318-0

[9] Hoffmann-Vold A-M, Allanore Y, Alves M, Brunborg C, Airó P, Ananieva LP, et al. Progressive interstitial lung disease in patients with systemic sclerosis-associated interstitial lung disease in the EUSTAR database. Ann Rheum Dis. 2021;80(2):219–27. 32988845 10.1136/annrheumdis-2020-217455 PMC7815627

[10] Goh NSL, Desai SR, Veeraraghavan S, Hansell DM, Copley SJ, Maher TM, et al. Interstitial Lung Disease in Systemic Sclerosis. Am J Respir Crit Care Med. 2008;177(11):1248–54. 18369202 10.1164/rccm.200706-877OC

[11] Wells AU. High-resolution computed tomography and scleroderma lung disease. Rheumatology (Oxford). 2008;47(Suppl 5):v59-61. 18784149 10.1093/rheumatology/ken271

[12] Bailey DL, Maisey MN, Townsend DW, Valk PE. Positron emission tomography. London: Springer; 2005.

[13] Volpi S, Ali JM, Tasker A, Peryt A, Aresu G, Coonar AS. The role of positron emission tomography in the diagnosis, staging and response assessment of non-small cell lung cancer. Ann Transl Med. 2018;6(5):95. 29666818 10.21037/atm.2018.01.25 PMC5890043

[14] Zerizer I, Tan K, Khan S, Barwick T, Marzola MC, Rubello D, Al-Nahhas A. Role of FDG-PET and PET/CT in the diagnosis and management of vasculitis. Eur J Radiol. 2010;73(3):504–9. 20172676 10.1016/j.ejrad.2010.01.021

[15] Jacquelin V, Mekinian A, Brillet PY, Nunes H, Fain O, Valeyre D, Soussan M. FDG-PET/CT in the prediction of pulmonary function improvement in nonspecific interstitial pneumonia. A Pilot Study Eur J Radiol. 2016;85(12):2200–5. 27842667 10.1016/j.ejrad.2016.10.001

[16] Uehara T, Takeno M, Hama M, Yoshimi R, Suda A, Ihata A, et al. Deep-inspiration breath-hold 18F-FDG-PET/CT is useful for assessment of connective tissue disease associated interstitial pneumonia. Mod Rheumatol. 2016;26(1):121–7. 25995034 10.3109/14397595.2015.1054099

[17] Bellando-Randone S, Tartarelli L, Cavigli E, Tofani L, Bruni C, Lepri G, et al. 18F-fluorodeoxyglucose positron-emission tomography/CT and lung involvement in systemic sclerosis. Ann Rheum Dis. 2019;78(4):577–8. 30337426 10.1136/annrheumdis-2018-213376

[18] Peelen DM, Zwezerijnen BGJC, Nossent EJ, Meijboom LJ, Hoekstra OS, Van der Laken CJ, Voskuyl AE. The quantitative assessment of interstitial lung disease with positron emission tomography scanning in systemic sclerosis patients. Rheumatology. 2020;59(6):1407–15. 31642912 10.1093/rheumatology/kez483 PMC7244784

[19] Ledoult E, Morelle M, Soussan M, Mékinian A, Béhal H, Sobanski V, et al. (18)F-FDG positron emission tomography scanning in systemic sclerosis-associated interstitial lung disease: a pilot study. Arthritis Res Ther. 2021;23(1):76. 33673861 10.1186/s13075-021-02460-8 PMC7936499

[20] Spierings J, van Rhenen A, Welsing PMW, Marijnissen ACA, De Langhe E, Del Papa N, et al. A randomised, open-label trial to assess the optimal treatment strategy in early diffuse cutaneous systemic sclerosis: the UPSIDE study protocol. BMJ Open. 2021;11(3): e044483. 33737437 10.1136/bmjopen-2020-044483 PMC7978271

[21] Boellaard R, Delgado-Bolton R, Oyen WJ, Giammarile F, Tatsch K, Eschner W, et al. FDG PET/CT: EANM procedure guidelines for tumour imaging: version 2.0. Eur J Nucl Med Mol Imaging. 2015;42(2):328–54. 25452219 10.1007/s00259-014-2961-x PMC4315529

[22] Boellaard R. Quantitative oncology molecular analysis suite: ACCURATE. J Nucl Med. 2018;59(supplement 1):1753-.

[23] Boktor RR, Walker G, Stacey R, Gledhill S, Pitman AG. Reference range for intrapatient variability in blood-pool and liver SUV for 18F-FDG PET. J Nucl Med. 2013;54(5):677–82. 23512357 10.2967/jnumed.112.108530

[24] Raghu G, Remy-Jardin M, Richeldi L, Thomson CC, Inoue Y, Johkoh T, et al. Idiopathic Pulmonary Fibrosis (an Update) and Progressive Pulmonary Fibrosis in Adults: An Official ATS/ERS/JRS/ALAT Clinical Practice Guideline. Am J Respir Crit Care Med. 2022;205(9):e18–47. 35486072 10.1164/rccm.202202-0399ST PMC9851481

[25] Hoffmann-Vold A-M, Aaløkken TM, Lund MB, Garen T, Midtvedt Ø, Brunborg C, et al. Predictive Value of Serial High-Resolution Computed Tomography Analyses and Concurrent Lung Function Tests in Systemic Sclerosis. Arthritis & Rheumatology. 2015;67(8):2205–12. 25916462 10.1002/art.39166

[26] Hansell DM, Bankier AA, MacMahon H, McLoud TC, Müller NL, Remy J. Fleischner Society: glossary of terms for thoracic imaging. Radiology. 2008;246(3):697–722. 18195376 10.1148/radiol.2462070712

[27] Moore OA, Goh N, Corte T, Rouse H, Hennessy O, Thakkar V, et al. Extent of disease on high-resolution computed tomography lung is a predictor of decline and mortality in systemic sclerosis-related interstitial lung disease. Rheumatology. 2012;52(1):155–60. 23065360 10.1093/rheumatology/kes289

[28] Goh NS, Hoyles RK, Denton CP, Hansell DM, Renzoni EA, Maher TM, et al. Short-Term Pulmonary Function Trends Are Predictive of Mortality in Interstitial Lung Disease Associated With Systemic Sclerosis. Arthritis Rheumatol. 2017;69(8):1670–8. 28426895 10.1002/art.40130

[29] Panopoulos S, Bournia V-K, Konstantonis G, Fragiadaki K, Sfikakis PP, Tektonidou MG. Predictors of morbidity and mortality in early systemic sclerosis: Long-term follow-up data from a single-centre inception cohort. Autoimmun Rev. 2018;17(8):816–20. 29885536 10.1016/j.autrev.2018.02.008

[30] Castiaux A, Van Simaeys G, Goldman S, Bondue B. Assessment of 18F-FDG uptake in idiopathic pulmonary fibrosis: influence of lung density changes. Eur J Hybrid Imaging. 2018;2(1):27.

[31] Motegi SI, Fujiwara C, Sekiguchi A, Hara K, Yamaguchi K, Maeno T, et al. Clinical value of (18) F-fluorodeoxyglucose positron emission tomography/computed tomography for interstitial lung disease and myositis in patients with dermatomyositis. J Dermatol. 2019;46(3):213–8. 30614031 10.1111/1346-8138.14758

[32] Inoue K, Okada K, Taki Y, Goto R, Kinomura S, Fukuda H. (18)FDG uptake associated with CT density on PET/CT in lungs with and without chronic interstitial lung diseases. Ann Nucl Med. 2009;23(3):277–81. 19319629 10.1007/s12149-009-0234-8

[33] Groves AM, Win T, Screaton NJ, Berovic M, Endozo R, Booth H, et al. Idiopathic pulmonary fibrosis and diffuse parenchymal lung disease: implications from initial experience with 18F-FDG PET/CT. J Nucl Med. 2009;50(4):538–45. 19289428 10.2967/jnumed.108.057901

[34] Justet A, Laurent-Bellue A, Thabut G, Dieudonné A, Debray MP, Borie R, et al. [(18)F]FDG PET/CT predicts progression-free survival in patients with idiopathic pulmonary fibrosis. Respir Res. 2017;18(1):74. 28449678 10.1186/s12931-017-0556-3 PMC5408423

[35] Nobashi T, Kubo T, Nakamoto Y, Handa T, Koyasu S, Ishimori T, et al. 18F-FDG Uptake in Less Affected Lung Field Provides Prognostic Stratification in Patients with Interstitial Lung Disease. J Nucl Med. 2016;57(12):1899–904. 27339874 10.2967/jnumed.116.174946

[36] Lee EY, Wong CS, Fung SL, Yan PK, Ho JC. SUV as an adjunct in evaluating disease activity in idiopathic pulmonary fibrosis - a pilot study. Nucl Med Commun. 2014;35(6):631–7. 24472818 10.1097/MNM.0000000000000083

[37] Maher TM, Wells AU, Laurent GJ. Idiopathic pulmonary fibrosis: multiple causes and multiple mechanisms? Eur Respir J. 2007;30(5):835–9. 17978154 10.1183/09031936.00069307

[38] Bergmann C, Distler JHW, Treutlein C, Tascilar K, Müller A-T, Atzinger A, et al. 68Ga-FAPI-04 PET-CT for molecular assessment of fibroblast activation and risk evaluation in systemic sclerosis-associated interstitial lung disease: a single-centre, pilot study. The Lancet Rheumatology. 2021;3(3):e185–94. 38279381 10.1016/S2665-9913(20)30421-5

[39] Adams H, Meek B, van de Garde EM, van Moorsel CH, Vugts DJ, Keijsers RG, Grutters JC. Altered splenic [(89)Zr]Zr-rituximab uptake in patients with interstitial lung disease not responding to rituximab: could this indicate a splenic immune-mediated mechanism? Am J Nucl Med Mol Imaging. 2020;10(4):168–77. 32929395 PMC7486548

[40] Adams H, van de Garde EM, van Moorsel CH, Vugts DJ, van Dongen GA, Grutters JC, Keijsers RG. [(89)Zr]Zr-rituximab PET/CT activity in patients with therapy refractory interstitial pneumonitis: a feasibility study. Am J Nucl Med Mol Imaging. 2019;9(6):296–308. 31976159 PMC6971479

[41] Lukey PT, Coello C, Gunn R, Parker C, Wilson FJ, Saleem A, et al. Clinical quantification of the integrin αvβ6 by [(18)F]FB-A20FMDV2 positron emission tomography in healthy and fibrotic human lung (PETAL Study). Eur J Nucl Med Mol Imaging. 2020;47(4):967–79. 31814068 10.1007/s00259-019-04586-z PMC7075837

[42] Branley HM, du Bois RM, Wells AU, Jones HA. PET scanning of macrophages in patients with scleroderma fibrosing alveolitis. Nucl Med Biol. 2008;35(8):901–9. 19026952 10.1016/j.nucmedbio.2008.10.001

[43] Grootjans W, Rietbergen DDD, van Velden FHP. Added Value of Respiratory Gating in Positron Emission Tomography for the Clinical Management of Lung Cancer Patients. Semin Nucl Med. 2022;52(6):745–58. 10.1053/j.semnuclmed.2022.04.006 35643531

[44] Tahari AK, Lodge MA, Wahl RL. Respiratory-gated PET/CT versus delayed images for the quantitative evaluation of lower pulmonary and hepatic lesions. J Med Imaging Radiat Oncol. 2014;58(3):277–82. 24438486 10.1111/1754-9485.12154 PMC4043855

[45] Manafi-Farid R, Askari E, Shiri I, Pirich C, Asadi M, Khateri M, et al. [18F]FDG-PET/CT Radiomics and Artificial Intelligence in Lung Cancer: Technical Aspects and Potential Clinical Applications. Semin Nucl Med. 2022;52(6):759–80. 35717201 10.1053/j.semnuclmed.2022.04.004

