# Supplementary material for: Quantitative 18F-FDG PET-CT can assess presence and extent of interstitial lung disease in early severe diffuse cutaneous systemic sclerosis
Source: Arthritis Res Ther. 2024 Dec 19;26:219. doi: 10.1186/s13075-024-03447-x (PMC11657652; doi:10.1186/s13075-024-03447-x)
Supplement: Supplementary file 1 — Supplementary Material 1. [file 13075_2024_3447_MOESM1_ESM.docx]

**SUPPLEMENTARY MATERIAL**

**Additional Data 1 – (Updated) Inclusion and exclusion criteria of the UPSIDE study
Inclusion criteria**

In order to be eligible to participate in this study, a subject must be eligible for HSCT treatment and therefore meet all of the following criteria:

1. Age between 18 and 65 years.
2. Fulfilling the 2013 ACR-EULAR classification criteria for SSc (appendix B).
Either: 3.1 or 3.2
3.1. Disease duration ≤ 3 years (from onset of first non-Raynaud’s symptoms) and diffuse cutaneous disease with
- Progressive skin involvement with a mRSS ≥ 15 (in a diffuse pattern: involvement of skin on the upper limbs, chest and/or abdomen) **and/or**- Major organ involvement as defined by either:

a. Clinically significant respiratory involvement =

i. DLCO and/or (F)VC ≤ 85% (of predicted) and evidence of interstitial lung disease on HRCT scan with clinically relevant obstructive disease and emphysema excluded.
ii. Patients with a DCLO and/or FVC > 85%, but with a progressive course of lung disease: defined as relative decline of >10% in FVC predicted and/or TLC predicted, or >15% in DLCO predicted and evidence of interstitial lung disease on HRCT scan with clinically relevant obstructive disease and emphysema excluded, within 12 months. Intercurrent infections excluded.

b. Clinically significant renal involvement =

i. New renal insufficiency (serum creatinine > upper limit of normal)
**and**

a. Persistent urinalysis abnormalities (proteinuria, haematuria, casts),
**and/or**

b. Microangiopathic haemolytic anaemia
**and/or**

c. Hypertension (two successive BP readings of either systolic ≥ 160 mm Hg or diastolic > 110 mm Hg, at least 12 hours apart); non-scleroderma related causes (e.g. medication, infection etc.) must be reasonably excluded.

c. Clinically significant cardiac involvement = any of the following criteria:

i. Reversible congestive heart failure,

ii. Atrial or ventricular rhythm disturbances such as atrial fibrillation or flutter, atrial paroxysmal tachycardia or ventricular tachycardia, 2nd or 3rd degree AV block,

iii. Pericardial effusion (not leading to hemodynamic problems), myocarditis; non-scleroderma related causes must have been reasonably excluded

3.2. Disease duration ≤ 1 year (from onset of first non-Raynaud’s symptoms) and diffuse cutaneous disease with mRSS ≥ 10 **and**

a. High risk ANA for organ based disease: ATA or ARA positivity **and/or**

b. Acute phase response (ESR > 25 mm/h and/or CRP > 10.0 mg/L)
4. Written Informed consent

**Exclusion criteria**
A potential subject who meets any of the following criteria will be excluded from participation in this study:

1. Pregnancy or unwillingness to use adequate contraception during study
2. Concomitant severe disease =

a) Respiratory: pulmonary hypertension: a resting mean pulmonary artery pressure (mPAP) > 25 mmHg (by right heart catheterisation); DLCO < 40% predicted or - respiratory failure as defined by the primary endpoint (see 8.1)

b) Renal: creatinine clearance < 40 ml/min (measured or estimated)

c) Cardiac: clinical evidence of refractory congestive heart failure; LVEF < 45% by cardiac echo or cardiac MR; chronic atrial fibrillation necessitating oral anticoagulation; uncontrolled ventricular arrhythmia; pericardial effusion with hemodynamic consequences (10)

d) Liver failure as defined by a sustained 3-fold increase in serum transaminase or bilirubin, or a Child- Pugh score C

e) Psychiatric disorders including active drug or alcohol abuse

f) Concurrent neoplasms or myelodysplasia, leading to exclusion of cyclophosphamide, mycophenolate mofetil or autologous stem cell transplantation in routine clinical practice. Patients with a history of malig-nancy of any organ system > 5 years ago, are allowed to participate.

g) Bone marrow insufficiency defined as leukocytopenia < 4.0 x 109/L, thrombocytopenia < 50 x 109/L, anaemia < 8 gr/dL, CD4+ T lymphopenia < 200 x 106/L

h) Uncontrolled hypertension (systolic blood pressure >150mmHg despite medication. *Blood pressure needs to be controlled prior to inclusion*

i) Uncontrolled acute or chronic infection, including HIV, HTLV-1,2 positivity, requiring hospitalization, IV antibiotics or leading to exclusion of cyclophosphamide, mycophenolate mofetil or autologous stem cell transplantation in routine clinical practice. The minimum time required between the infection and enrolment is ideally 7 days after complete recovery. Latent infections such as tuberculosis and hepatitis need to be treated prior to inclusion and according to local protocols. *Infection needs to be treated/controlled prior to inclusion.*

j) ZUBROD-ECOG-WHO Performance Status Scale > 2 (Appendix C)

k) Known hypersensitivity to any of the study drug constituents

3. Previous treatments with immunosuppressants > 12 months including MMF, methotrexate, azathioprine, rituximab, tocilizumab, glucocorticosteroids.

4. Previous treatments with TLI, TBI or alkylating agents including CYC.

5. Significant exposure to bleomycin, tainted rapeseed oil, vinyl chloride, trichlorethylene or silica.

6. Eosinophilic myalgia syndrome; eosinophilic fasciitis.

7. Poor compliance of the patient as assessed by the referring physicians.

**Additional Data 2 - Methods of PET/CT quantification**


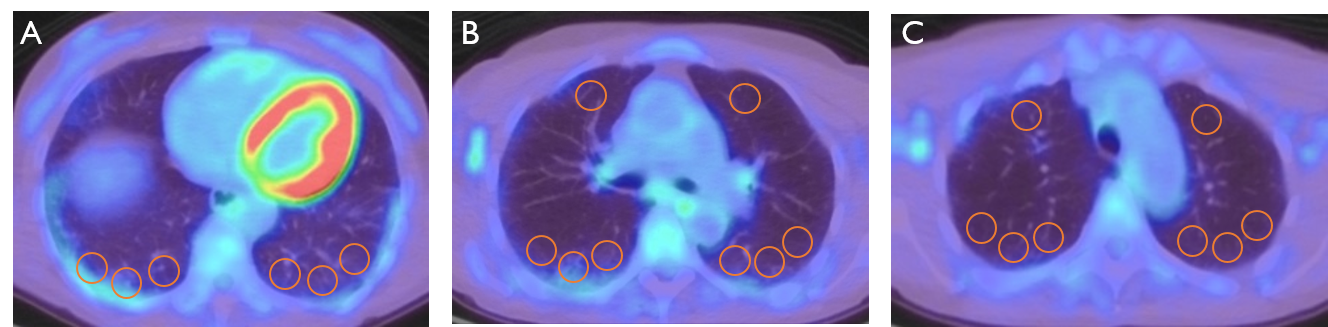


**Locations of Volumes of Interest (VOIs)**In each patient, 22 VOIs were drawn in the transversal slides of different lung areas. VOIs were checked in sagittal and coronal views to verify correct placement: **(A)** basal (6 dorsal) VOIs located at 2cm from the diaphragm, **(B)** mid-level (6 dorsal, 2 ventral) VOIs located just below the carina and **(C)** apical (6 dorsal, 2 ventral) VOIs located at the upper aortic arch.

**Additional Table 1 - Radiologic findings and mean SUVmax in the basal, mid-level and apical lung fields in SSc-ILD.**

|  | Basal (dorsal)  n (%) | Mid-level (dorsal) n (%) | Mid-level (ventral) n (%) | Apical (dorsal) n (%) | Apical (ventral) n (%) |
| --- | --- | --- | --- | --- | --- |
| Normal | 1 (3.6%) | 20 (71.4%) | 25 (89.3%) | 26 (92.9%) | 24 (86.7%) |
| GGO | 20 (71.4%) | 4 (14.3%) | 3 (10.7%) | 2 (7.1%) | 4 (4.3%) |
| GGO + Reticulation | 7 (25.0%) | 3 (10.7%) | 0 (0.0%) | 0 (0.0%) | 0 (0.0%) |
| Reticulation | 0 (0.0%) | 1 (3.6%) | 0 (0.0%) | 0 (0.0%) | 0 (0.0%) |

|  | Basal (dorsal)  SUVmax | Mid-level (dorsal) SUVmax | Mid-level (ventral) SUVmax | Apical (dorsal) SUVmax | Apical (ventral) SUVmax |
| --- | --- | --- | --- | --- | --- |
| Normal | 0.70 (0.07) | 0.74 (0.26) | 0.44 (0.14) | 0.59 (0.20) | 0.46 (0.16) |
| GGO | 0.98 (0.23) | 0.98 (0.26) | 0.45 (0.05) | 0.88 (0.26) | 0.54 (0.10) |
| GGO + Reticulation | 1.23 (0.32) | 0.87 (0.26) | - | - | - |
| Reticulation | - | 1.00 (0.28) | - | - | - |

Data are presented as number (%) and mean (standard deviation).
GGO = Ground-Glass Opacities; SUVmax = maximum Standardized Uptake Value

**Additional Figure 1 - Quantitative pulmonary uptake of ^18^F-FDG in patients with SSc-ILD, SSc without ILD and controls.**
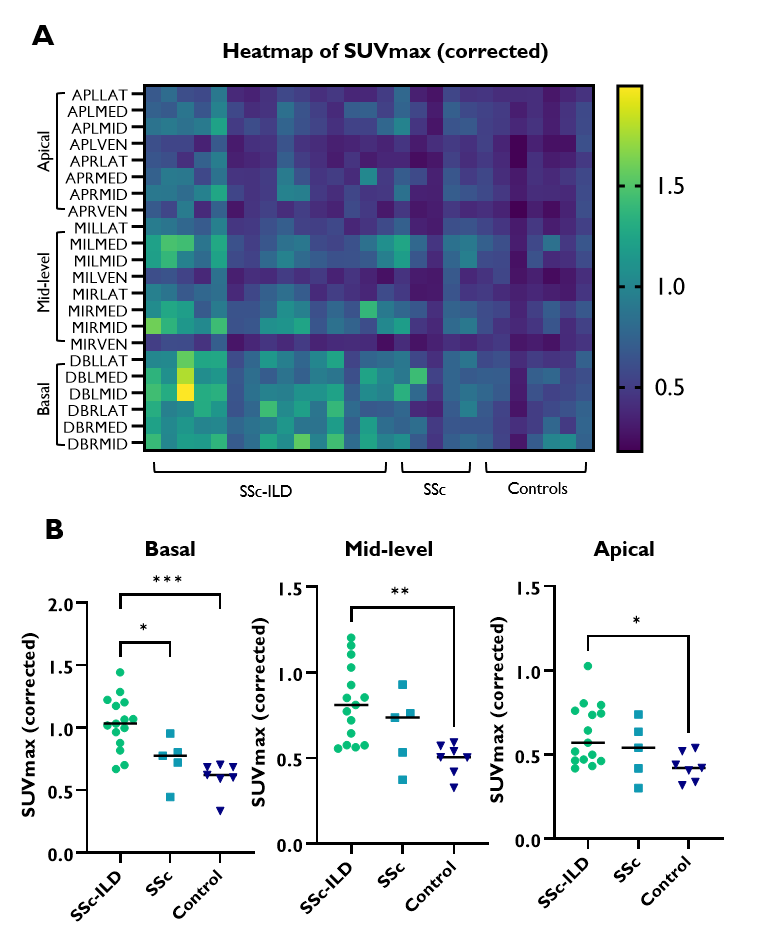


A. Heatmap of SUVmax (corrected) in individual VOIs across SSc-ILD, SSc without ILD and controls B. Pooled SUVmax (corrected) in the 6 dorsobasal VOIs, 6 mid-level dorsal VOIs and 6 apical dorsal VOIs. * p < 0.05 ** p < 0.002 *** p <0.001

ILD = Interstitial Lung Disease; SSc = Systemic Sclerosis; SUVmax = maximum Standardized Uptake Value; VOI = Volume of Interest #AP = Apical; MI = Mid-level; DB = Dorsobasal; L = Left; R = Right; LAT = Lateral; MED = Medial; MID = Middle; VEN = Ventral

**Additional Figure 2** - **Relation of ^18^F-FDG uptake in the dorsobasal lung fields with ILD extent and pulmonary function test parameters in SSc-ILD.**


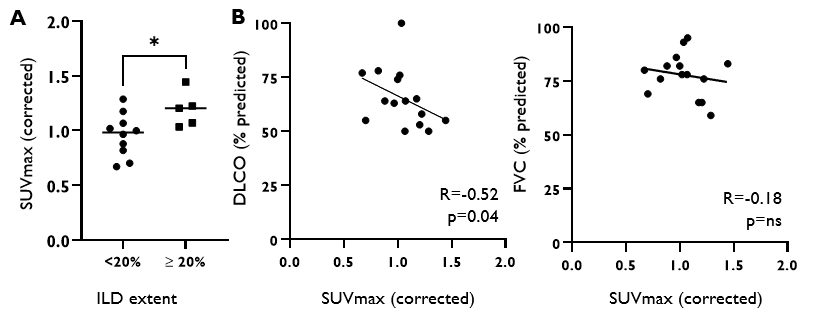


A. Pulmonary uptake of ^18^F-FDG in SSc-ILD, stratified by ILD extent on HRCT (<20% and ≥20%) B. Correlation between pulmonary uptake of ^18^F-FDG and pulmonary function test parameters in SSc-ILD * p < 0.05

DLCO = Diffusing capacity of the Lungs for Carbon Monoxide; FVC = Forced Vital Capacity; HRCT = High Resolution Computed Tomography; ILD = Interstitial Lung Disease; SSc = Systemic Sclerosis; SUVmax = max Standardized Uptake Value
